# Ferroelectric Organic–Inorganic Hybrid Ammonium Halogenobismuthate(III) for Piezoelectric Energy Harvesting

**DOI:** 10.1021/acs.inorgchem.4c00908

**Published:** 2024-05-03

**Authors:** Namonarayan Meena, Supriya Sahoo, Nilotpal Deka, Jan K. Zaręba, Ramamoorthy Boomishankar

**Affiliations:** † Department of Chemistry, Indian Institute of Science Education and Research, Pune, Dr. Homi Bhabha Road, Pune 411008, India; ‡ Centre for Energy Science, Indian Institute of Science Education and Research, Pune, Dr. Homi Bhabha Road, Pune 411008, India; § Institute of Advanced Materials, Wrocław University of Science and Technology, 50-370 Wrocław, Poland

## Abstract

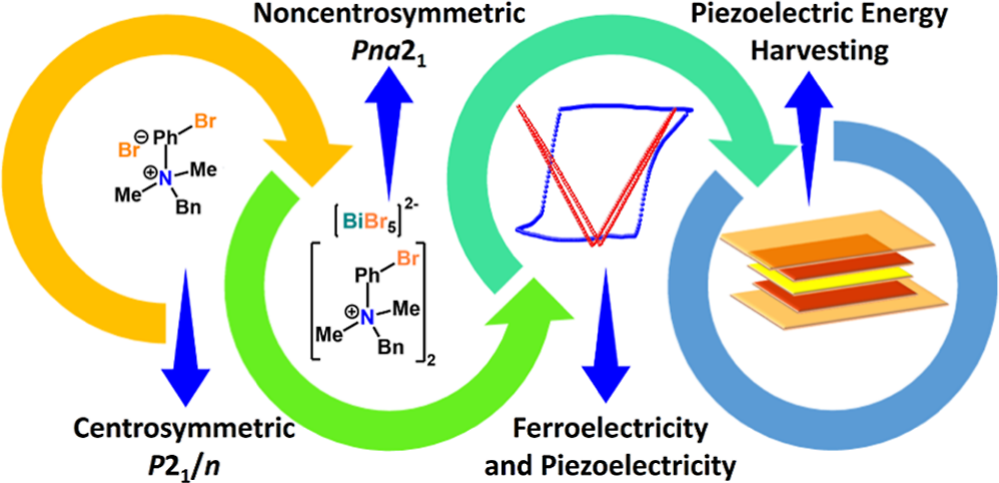

Halogenobismuthate(III) compounds are of recent interest because of their low toxicity and distinct electrical properties. The utility of these materials as ferroelectrics for piezoelectric energy harvesters is still in its early stages. Herein, we report a hybrid ammonium halogenobismuthate(III) **[BP**_**Br**_**DMA]**_**2**_**·[BiBr**_**5**_**]**, crystallizing in a ferroelectrically active polar noncentrosymmetric *Pna*2_1_ space group. Its noncentrosymmetric structure was confirmed by the detection of the second harmonic generation response. The ferroelectric *P*–*E* hysteresis loop measurements on the thin film sample of **[BP**_**Br**_**DMA]**_**2**_**·[BiBr**_**5**_**]** gave a saturation polarization (*P*_s_) of 5.72 μC cm^–2^. The piezoresponse force microscopy analysis confirmed its ferroelectric and piezoelectric nature, showing characteristic domain structures and signature hysteresis and butterfly loops. The piezoelectric energy harvesting attributes of **[BP**_**Br**_**DMA]**_**2**_**·[BiBr**_**5**_**]** were further probed on its polylactic acid (PLA) composites. The 15 wt % **[BP**_**Br**_**DMA]**_**2**_**·[BiBr**_**5**_**]**-PLA polymer composite resulted in a high output voltage of 26.2 V and power density of 15.47 μW cm^–2^. The energy harvested from this device was further utilized for charging a 10 μF capacitor within 3 min.

## Abstract 2

A new room-temperature ferroelectric Halogenobismuthate(III) **[BP**_**Br**_**DMA]**_**2**_**·[BiBr**_**5**_**]** was synthesized and studied for its excellent ferroelectric and piezoelectric properties. The polylactic acid composites of **[BP**_**Br**_**DMA]**_**2**_**·[BiBr**_**5**_**]** further demonstrated its utility for piezoelectric energy harvesting applications with high open circuit peak-to-peak voltage and peak power density values.

## Introduction

Technological applications increasingly rely on multifunctional materials due to their unique physical and chemical properties.^1^ Within this domain, ferroelectrics, a subset of dielectric materials, have garnered significant attention for their potential in various applications, including ferroelectric random-access memories, microelectromechanical devices, wireless electronics, transducers, wearable memory devices, robotics, energy harvesting, etc.^2−5^ For instance, the multifunctional nature of ferroelectric materials is evident from their utility for piezoelectric energy harvesting, wherein a sizable ferroelectric polarization aids in the efficient conversion of mechanical energy into electrical outputs.^6−8^ Over the years, ceramic oxide materials such as barium titanate, lithium niobate, lead titanate, lead zirconate titanate (PZT), zinc oxide, etc., have been the focus of extensive research due to their excellent ferroelectric and piezoelectric attributes.^9−13^ However, their brittleness and toxic metal content limit their suitability for low-power, wearable electronics.^10^ On the other hand, semicrystalline organic polymers, such as polyvinylidene fluoride and its copolymers, offer a flexible, metal-free alternative, showing ferroelectricity and potential for device applications.^14^ However, challenges exist related to poor crystallinity and phase purity, requiring high-voltage poling and external additives to achieve the desired properties.^15^ Therefore, the selection of materials for ferroelectricity and energy harvesting is critical and necessitates a balance between the attributes of polymers and ceramic oxides.

In this regard, organic–inorganic hybrid materials have recently shown great potential for their easy synthesis, processability, flexibility, stability, and high polarization attributes.^16−18^ These highly crystalline materials have extensively been studied for optoelectronics and solar cells, benefiting from tunable band gap, long diffusion length, high carrier mobility, strong optical absorption, and high solution processability.^19,20^ In the domain of ferroelectricity, significant efforts have been directed toward the design of lead-free hybrid organic–inorganic materials supported by ammonium- and phosphonium cations.^21−23^ However, the synthesis of suitable ferroelectric two-component materials has its own challenges because of their requirement for noncentrosymmetric polar space groups.^24^ Since structural asymmetry is a cumulative effect of the overall composition, noncentrosymmetry in two-component systems can be induced by modifications to both the cationic and anionic components of the hybrid assembly.^24,25^

Halobismuthates(III), represented by the formula A_*a*_Bi_*b*_X_3*b*+*a*_, (wherein A denotes organic cations and X stands for halogens), along with the closely related class of haloantimonates(III), have emerged as materials of significant interest due to their unique blend of advantageous characteristics.^26−30^ These include straightforward synthesis and processing techniques, cost-effectiveness, and useful electrical properties. Halobismuthates(III) exhibiting polar characteristics are particularly relevant for applications due to their ferroelectric properties, which, however, are confined to a select few stoichiometric compositions, namely A_2_BiX_5_,^31−35^ A_3_Bi_2_X_9_,^36−38^ and A_5_Bi_2_X_11_.^39^ Among these compositions, A_2_BiX_5_-type molecular ferroelectrics have shown distinctly good ferroelectric properties. However, a common denominator for materials such as (C_2_H_5_NH_3_)_2_[BiBr_5_]^40^ (phase I: *Aeam* (160 K), phase II: *Pca*2_1_ (120 K), phase III: *Aea*2), (MV)[BiBr_5_]^41^ (MV = methylviologen) (240 K), (C_2_H_5_NH_3_)_2_[BiCl_5_]^33^ (190 K) etc., is that they exhibit ferroelectric-paraelectric (*T*_c_) phase transitions below room temperature, hindering their practical utility. To overcome this challenge and attain high *T*_c_ ferroelectrics, the “quasi-spherical theory” has been proposed by Xiong et al. as one of the design approaches for realizing ferroelectricity.^42^ The quasi-spherical theory proposes that the modifications to spherical molecules can lead to the synthesis of cations with frozen dipoles, thus resulting in high *T*_c_ ferroelectric materials suitable for room temperature applications.

Guided by this theory, we have successfully designed and synthesized a new high-*T*_c_ ferroelectric material by partially substituting bulkier heteroleptic groups (benzyl, bromophenyl) at the ammonium center, while retaining two small methyl substituents. Remarkably, despite the presence of a *C*_*s*_ symmetric [(*p*-BrPh)BnNMe_2_]^+^ cation [(*p*-BrPh)BnNMe_2_ = *N*-benzyl-4-bromo-*N*,*N*-dimethylbenzenaminium: further abbreviated as **BP**_**Br**_**DMA**], its bromide salt **BP**_**Br**_**DMA·Br** crystallizes in the centrosymmetric monoclinic space group *P*2_1_/*n*. However, the introduction of the bulky multiatomic [BiBr_5_]^2–^ anionic network facilitates the crystallization of **[BP**_**Br**_**DMA]**_**2**_**·[BiBr**_**5**_**]** with a vertex sharing bismuth bromide octahedral anionic network in noncentrosymmetric polar *Pna*2_1_ space group as evidenced by second harmonic generation (SHG) and X-ray diffraction analysis. The *P*–*E* hysteresis loop measurements on the thin film sample of **[BP**_**Br**_**DMA]**_**2**_**·[BiBr**_**5**_**]** resulted in a rectangular hysteresis loop with saturation polarization of 5.72 μC cm^–2^. The microscopic polarization properties of **[BP**_**Br**_**DMA]**_**2**_**·[BiBr**_**5**_**]** were evident from the piezoresponse force microscopy (PFM) studies, which gave the characteristic phase-bias hysteresis and amplitude-bias butterfly loops. The piezoelectric nanogenerator devices fabricated from **[BP**_**Br**_**DMA]**_**2**_**·[BiBr**_**5**_**]**, embedded in polylactic acid (PLA) polymer, showed a maximum output voltage of 26.2 V and power density of 15.47 μW cm^–2^ for a 15 wt % composite device. Further, the practical utility of this device was demonstrated by efficiently charging a 10 μF capacitor, achieving a charge accumulation of 14 μC. These studies shed light on a systematic approach toward the design of lead-free hybrids for ferroelectric applications.

## Results and Discussion

### Syntheses, Spectra, Crystal Structure, and Hirshfeld Surface Analyses

**Figure 1:**
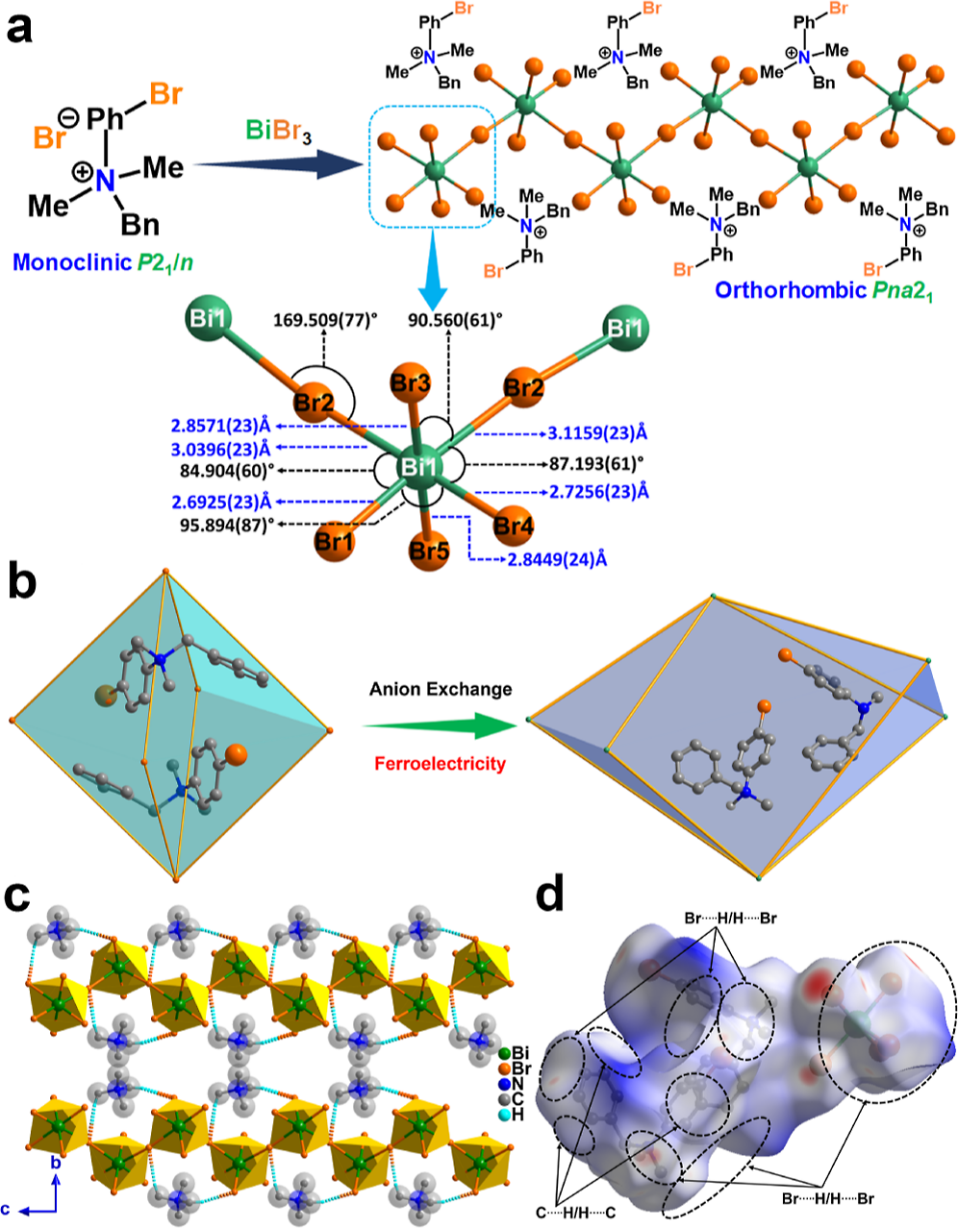
(a) Schematic showing the strategy of obtaining the polar hybrid of **[BP**_**Br**_**DMA]**_**2**_**·[BiBr**_**5**_**]** from the nonpolar **BP**_**Br**_**DMA·Br**. (b) Fragment of the packing structures of **BP**_**Br**_**DMA·Br** and **[BP**_**Br**_**DMA]**_**2**_**·[BiBr**_**5**_**]** showing the transformation of the cationic ammonium groups from the centrosymmetric (former) to the noncentrosymmetric (latter) setting. (c) H-bonding interactions present in **[BP**_**Br**_**DMA]**_**2**_**·[BiBr**_**5**_**]** in the crystal packing (the –Ph, –Bn groups are omitted for clarity). (d) *d*_norm_-mapped HS showing different interactions present in **[BP**_**Br**_**DMA]**_**2**_**·[BiBr**_**5**_**]**.

The precursor ammonium bromide salt **BP**_**Br**_**DMA·Br** was obtained as rod-shaped crystals from the reaction mixture of benzyl bromide and 4-bromo-*N*,*N*-dimethylaniline in methanol (Scheme S1). It crystallizes in the centrosymmetric monoclinic *P*2_1_/*n* space group (Figures 1a,b and S1–S3 and Table S1). The preparation of the hybrid assembly of **[BP**_**Br**_**DMA]**_**2**_**·[BiBr**_**5**_**]** involves the addition of Bi_2_O_3_ in HBr to a solution of **BP**_**Br**_**DMA·Br** in HBr (Scheme S2). The resultant precipitate was filtered and recrystallized from a mixture of acetone and acetonitrile to yield the yellow rod-shaped crystals of **[BP**_**Br**_**DMA]**_**2**_**·[BiBr**_**5**_**]**.

The single crystal X-ray diffraction (SCXRD) analyses revealed that the compound **[BP**_**Br**_**DMA]**_**2**_**·[BiBr**_**5**_**]** crystallizes in the polar noncentrosymmetric *Pna*2_1_ space group at 120 and 298 K (Figures 1a,b and S4 and S5 and Table S1). Figure 1b illustrates the arrangement of **BP**_**Br**_**DMA** cations in the respective structures of **BP**_**Br**_**DMA·Br** and **[BP**_**Br**_**DMA]**_**2**_**·[BiBr**_**5**_**]**, where the respective presence and absence of inversion centers in the vicinity of the ammonium cations can be visualized. This is evidence for the strategic replacement of spherical Br^–^ anion with a vertex-shared framework of [BiBr_5_]^2–^ anions gives rise to a polar material of composition **[BP**_**Br**_**DMA]**_**2**_**·[BiBr**_**5**_**]** supported by the *C*_*s*_ symmetric **BP**_**Br**_**DMA** cations.

The asymmetric unit of **[BP**_**Br**_**DMA]**_**2**_**·[BiBr**_**5**_**]** consists of two ammonium cations and one [BiBr_5_]^2–^ motif, which assemble into a vertex-sharing [BiBr_5_]^2–^ 1D-octahedral chain. The two bridging vertices of each octahedron adopt a cis configuration with an average Bi–Br–Bi angle of 169.51(8)° (Figure 1a bottom). The anionic chain, including the coordination polyhedra, can be represented by the symbol ∞^1^[Bi^*O*^Br^2*n*^Br_4_]^2–^ as found in the structure of many reported polymeric [BiBr_5_]^2–^ and [MX_5_]^2–^-based ionic compounds.^43−47^ The net result is the formation of anionic [BiBr_5_]^2–^ chains with a zigzag arrangement, in which the ammonium cations are located in the pockets found between the octahedral units. The presence of bulky substituents on the ammonium center may contribute to the distortions observed in the octahedral chain of [BiBr_5_]^2–^. Indeed, the Bi–Br bond lengths in the network span from 2.6925(23) to 3.1159(23) Å, with the longest bonds corresponding to the Br atoms involved in the cis-connected network. By contrast, Br atoms trans to the long connection display the shortest terminal Bi–Br bonds. The remaining two terminal bonds are intermediate between these two distances.

A closer look at the structure of **[BP**_**Br**_**DMA]**_**2**_**·[BiBr**_**5**_**]** reveals the presence of weak H-bonding C–H···Br interactions, mediated by the methyl and methylene groups of the cations and the bromine atoms of the [BiBr_5_]^2–^ anions (Table S2). In fact, the preferred selection of cis-connection over the trans connection for the [BiBr_5_]^2–^ chains could be attributed to the bulkiness of the cation as well the presence of C–H···Br interactions. The presence of sterically hindered groups such as –BrPh and –Bn groups in the ammonium center orients the cation in such a way that the less hindered groups such as –Me and –CH_2_ come in close contact with the inorganic BiBr_5_ chain through C–H···Br interactions. Each cation interacts with two octahedra, in which the terminal Br atom (Br5) is bonded to the methylene proton (H2B) and the bridging Br2 atom interacts with the methyl proton (H21B) (Figures 1c and S6 and Table S2). To obtain further insights into the different types of interactions present in **[BP**_**Br**_**DMA]**_**2**_**·[BiBr**_**5**_**]**, Hirshfeld surface (HS) analyses were carried out using the Crystal Explorer software on the 120 K SCXRD structure (Figures 1d and S7 and Table S3). The surfaces depicted in red color on the *d*_norm_ surface plot imply the short interactions experienced by the molecule, while the blue-colored surfaces relate to the weak interactions in the molecule. The decomposition of 2D fingerprint plots (histograms of *d*_*i*_ plotted vs *d*_*e*_ values) into respective contacts yields the percentages of various interactions present in **[BP**_**Br**_**DMA]**_**2**_**·[BiBr**_**5**_**]** (Figure S8 and Table S4). Indeed, one sees that the C–H···Br contacts are the most prominent ones, contributing 53% of the overall interactions.

The bulk-phase purity of compound **[BP**_**Br**_**DMA]**_**2**_**·[BiBr**_**5**_**]** was confirmed from powder X-ray diffraction (PXRD) analyses. The observed PXRD profiles from the experiment match well with the peaks simulated from the 298 K SCXRD data (Figure S9). The thermogravimetric analysis plot of **[BP**_**Br**_**DMA]**_**2**_**·[BiBr**_**5**_**]** confirmed its stability up to 373 K (Figure S10). After 373 K, the step-like weight loss of 5.7% corresponds to the loss of solvated molecules (diffuse water and methanol used for the synthesis). Upon further heating, a weight loss of 31.7% corresponds to the decomposition of (*p*-BrPh)NMe_2_, followed by a 14.4% weight loss for Bn decomposition. The final weight loss of 48.1% is due to the decomposition of inorganic anion BiBr_5_. To investigate the presence of any structural changes (morphotropic phase boundary/phase transition) in **[BP**_**Br**_**DMA]**_**2**_**·[BiBr**_**5**_**]**, differential scanning calorimetry measurements were performed. However, no phase transition could be detected for **[BP**_**Br**_**DMA]**_**2**_**·[BiBr**_**5**_**]** in the temperature range of 223–373 K.

The optical properties of **[BP**_**Br**_**DMA]**_**2**_**·[BiBr**_**5**_**]** were studied in the solid-state diffuse reflectance UV–visible spectroscopy, which gave a pronounced absorption peak at 375 nm. From the Tauc plot, the bandgap of **[BP**_**Br**_**DMA]**_**2**_**·[BiBr**_**5**_**]** was measured to be 2.91 eV (Figure S11). This value is in line with the bandgaps observed for numerous organic–inorganic hybrid materials^48,49^ and is significantly lower than those typically reported for ceramic materials.

### Nonlinear Optical, Dielectric, Ferroelectric, and PFM Studies

**Figure 2:**
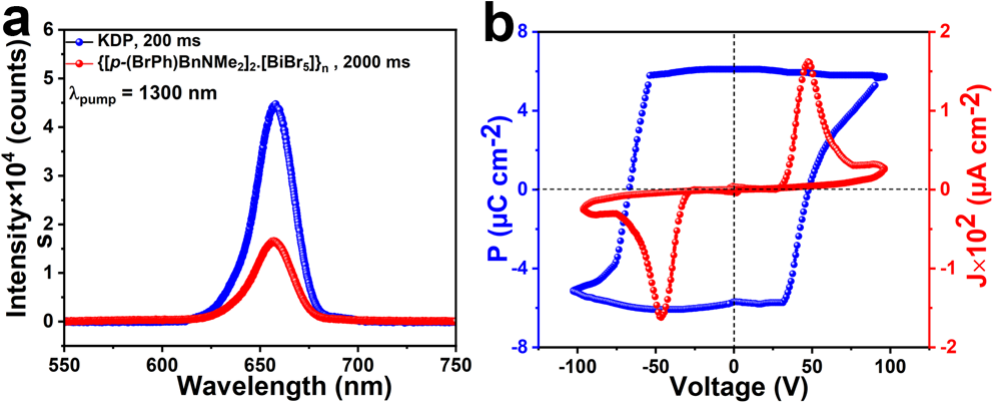
(a) Comparison of SHG emission profiles of **[BP**_**Br**_**DMA]**_**2**_**·[BiBr**_**5**_**]** with respect to the standard KDP sample. (b) *P*–*E* hysteresis loop for **[BP**_**Br**_**DMA]**_**2**_**·[BiBr**_**5**_**]**.

The noncentrosymmetric nature of **[BP**_**Br**_**DMA]**_**2**_**·[BiBr**_**5**_**]** is further validated by SHG measurements (Figure 2a). Under 1300 nm femtosecond laser irradiation, the size-graded (250–175 μm) powder samples of **[BP**_**Br**_**DMA]**_**2**_**·[BiBr**_**5**_**]** present a relative SHG efficiency of 0.039 with respect to the reference potassium dihydrogen phosphate (KDP) sample of approximately the same size at room temperature.

Furthermore, the dielectric nature of **[BP**_**Br**_**DMA]**_**2**_**·[BiBr**_**5**_**]** was investigated in a temperature range of 298–378 K. The real part of the dielectric permittivity (ε′) was found to be 9.5 at room temperature and 100 kHz frequency (Figure S12a). The temperature dependence of ε′ for **[BP**_**Br**_**DMA]**_**2**_**·[BiBr**_**5**_**]** showed an increasing trend near the decomposition temperature, which can be attributed to the presence of thermally activated free charge carriers (Figure S12a). The absence of any heat anomalies in the temperature dependence of ε′ can be attributed to the stable charge-separated structure of **[BP**_**Br**_**DMA]**_**2**_**·[BiBr**_**5**_**]**, aided by effective C–H···Br H-bonding interactions between the cations and the anionic chain. The frequency dependence of ε′ revealed a decrease in values with increasing frequencies from 10 to 10^5^ Hz (Figure S12b). The higher ε′ values at lower frequencies indicate the involvement of all types of polarization mechanisms (electronic, ionic, orientational, and space charge polarization) in **[BP**_**Br**_**DMA]**_**2**_**·[BiBr**_**5**_**]**. The plots of dielectric loss factors (tan δ) as a function of temperature and frequency showed a lower dissipation in polarization as heat, corroborating the true dielectric nature of this material (Figure S13).

The structure of **[BP**_**Br**_**DMA]**_**2**_**·[BiBr**_**5**_**]** exhibits a point group symmetry of *C*_2*v*_, indicative of its potential for ferroelectric behavior. Ferroelectric properties of **[BP**_**Br**_**DMA]**_**2**_**·[BiBr**_**5**_**]** were investigated through the analysis of thin films prepared by drop-casting on indium tin oxide (ITO)-coated glass substrates (Figure S14). A symmetrical rectangular *P*–*E* hysteresis loop with saturation polarization (*P*_s_) of 5.72 μC cm^–2^ has been recorded for its film at room temperature. The leakage current density plot as a function of applied voltage shows low leakage characteristics with peaks corresponding to its coercive voltages, confirming the ferroelectric nature of the obtained *P*–*E* loop. The values are comparable with other hybrid materials based on bismuth(III) halide anions.^31,38,50,51^

**Figure 3:**
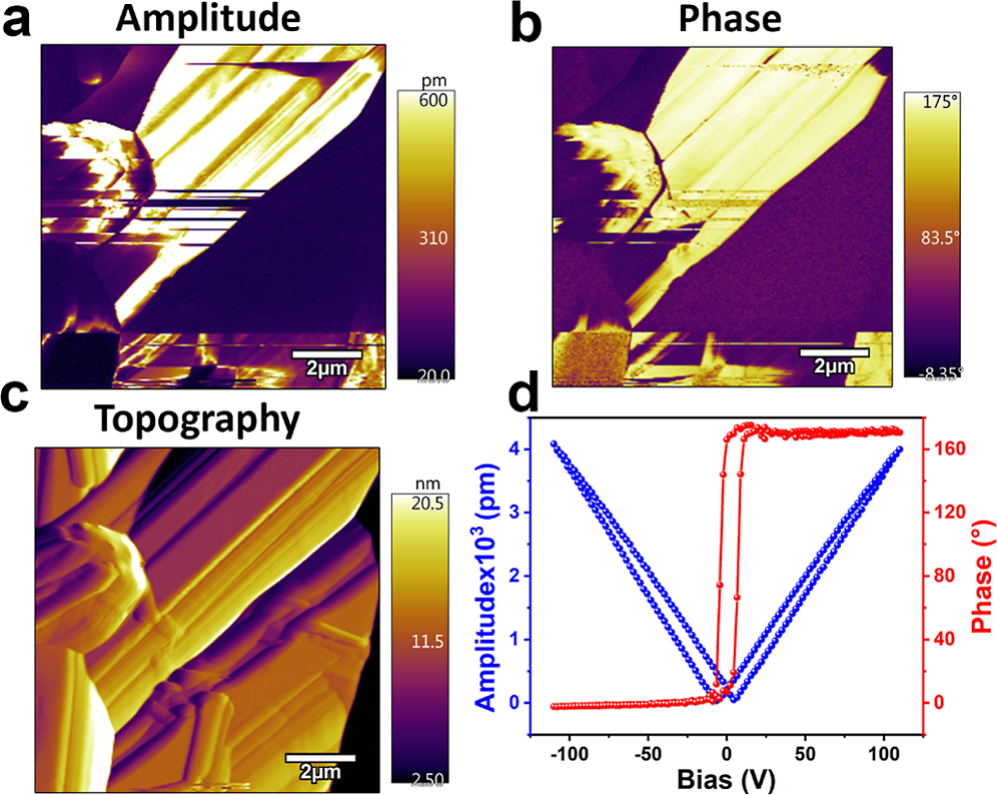
PFM-derived (a) amplitude image (b) phase image (c) topography of **[BP**_**Br**_**DMA]**_**2**_**·[BiBr**_**5**_**]**. (d) Its amplitude-bias “butterfly” (blue) and phase-bias “hysteresis” (red) loops.

The microscopic polarization characteristics of **[BP**_**Br**_**DMA]**_**2**_**·[BiBr**_**5**_**]** were further verified by vertical piezoresponse force microscopic studies on its drop-casted thin film onto an ITO-coated glass substrate. From these measurements, the amplitude- and phase-contrast images for the ferro- and piezoelectric domains of **[BP**_**Br**_**DMA]**_**2**_**·[BiBr**_**5**_**]** were extracted. The amplitude image shows the presence of multiple domains, while the phase image shows the 180° alignment of adjacent domains, indicating the multiaxial polarization of the material (Figure 3a,b). Furthermore, PFM spectroscopic analyses, by applying an additional DC voltage of ±120 V, were performed at a single point on the topographic surface of **[BP**_**Br**_**DMA]**_**2**_**·[BiBr**_**5**_**]**. These measurements gave the signature phase-bias hysteresis and amplitude-bias butterfly loops, confirming the switchable nature of its domain structure and polarization (Figure 3c,d). Such characteristic loops were noticed during the off-state PFM response measurements as well, supporting its ferro- and piezoelectric behavior (Figure S15). From the slopes of the butterfly loop, the converse piezoelectric coefficient (*d*_33_) of **[BP**_**Br**_**DMA]**_**2**_**·[BiBr**_**5**_**]** was calculated to be 36–39 pm V^–1^.

### Piezoelectric Energy Harvesting Studies

**Figure 4:**
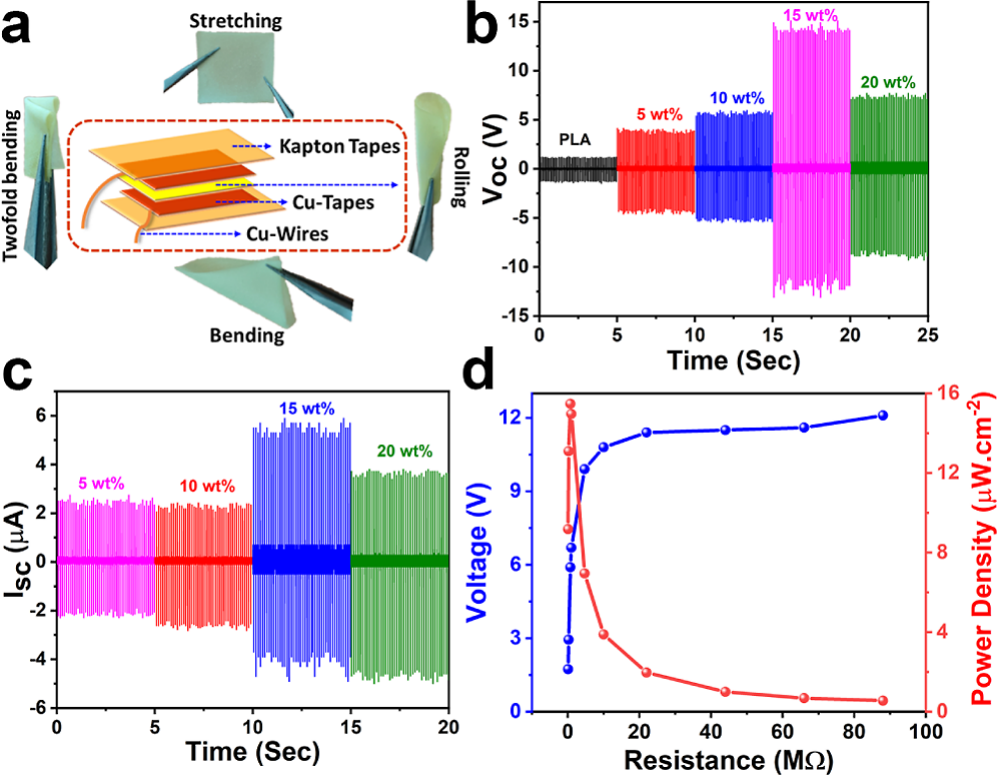
(a) Schematic showing the PENG device assembly and the pictures of the 15 wt % **[BP**_**Br**_**DMA]**_**2**_**·[BiBr**_**5**_**]**-PLA composite film showing its flexibility toward various stretching, rolling and bending motions. (b) Open-circuit peak-to-peak voltages obtained for **[BP**_**Br**_**DMA]**_**2**_**·[BiBr**_**5**_**]**-PLA composite devices. (c) Short-circuit current profiles for **[BP**_**Br**_**DMA]**_**2**_**·[BiBr**_**5**_**]**-PLA composite devices. (d) Resistance-dependent voltage drop for the 15 wt % **[BP**_**Br**_**DMA]**_**2**_**·[BiBr**_**5**_**]**-PLA composite device and the calculated power density across the load resistances.

Spurred by the recent developments in the use of hybrid ferroelectric materials as piezoelectric nanogenerators (PENGs), we set out to probe **[BP**_**Br**_**DMA]**_**2**_**·[BiBr**_**5**_**]** for the piezoelectric energy harvesting studies. To make flexible devices, composite films were prepared in chloroform solution using PLA polymer and **[BP**_**Br**_**DMA]**_**2**_**·[BiBr**_**5**_**]** in various (5, 10, 15, and 20) weight percentages (wt %) (Figure S16). The PXRD profiles of **[BP**_**Br**_**DMA]**_**2**_**·[BiBr**_**5**_**]**-PLA composites confirmed the crystalline nature of the embedded ferroelectric compound in the PLA matrix, which also revealed an enhancement in peak intensity correlating with the increased loading of the ferroelectric crystallites (from 5 to 20 wt %) in the composites (Figure S17). The obtained composite films of **[BP**_**Br**_**DMA]**_**2**_**·[BiBr**_**5**_**]**-PLA were found to exhibit excellent flexibility toward various mechanical distortions such as stretching, bending, rolling, and 2-fold bending operations (Figure 4a). The field-effect scanning electron microscopy technique was used to further visualize their structural morphologies, which showed an even distribution of the ferroelectric particles up to 15 wt % **[BP**_**Br**_**DMA]**_**2**_**·[BiBr**_**5**_**]**-PLA composites, while particle aggregation was observed for 20 wt % composite film (Figure S18). The device assembly was completed by placing conducting copper adhesive tapes as electrodes, copper wire as lead contacts, and Kapton tapes for external insulation (Figure 4a).

The piezoelectric energy harvesting measurements were performed at an external load of 21 N and at a constant frequency of 10 Hz for all **[BP**_**Br**_**DMA]**_**2**_**·[BiBr**_**5**_**]**-PLA composite devices. For the batch of **[BP**_**Br**_**DMA]**_**2**_**·[BiBr**_**5**_**]**-PLA composite devices, the maximum open circuit peak-to-peak voltage was obtained to be 26.2 V for the 15 wt % device. Whereas lower *V*_PP_ values of 8.5, 11.1, and 17.1 V were observed for the rest of the 5, 10, and 20 wt % **[BP**_**Br**_**DMA]**_**2**_**·[BiBr**_**5**_**]**-PLA devices, respectively (Figures 4b and S19). The reduction in open circuit voltages for the higher 20 wt % **[BP**_**Br**_**DMA]**_**2**_**·[BiBr**_**5**_**]**-PLA composite device could be attributed to the randomization of dipoles due to particle agglomeration, typically termed as Maxwell–Wagner–Sillar polarization.^52^ The open circuit output voltage for a neat PLA device yielded a modest value of 2.04 V owing to its weak piezoelectric nature. Thus, the much higher output voltages measured for the **[BP**_**Br**_**DMA]**_**2**_**·[BiBr**_**5**_**]**-PLA composite devices could be attributed to the presence of ferroelectric **[BP**_**Br**_**DMA]**_**2**_**·[BiBr**_**5**_**]** crystallites in the PLA matrix. The short circuit peak-to-peak currents (*I*_PP_ = *V*_PP_/*R*) were calculated by attaching a load resistance of 1 MΩ across the circuit and measuring the voltage drop. The calculated currents were found to be 4.59, 5.03, 10.63, and 8.29 μA, respectively, for the 5, 10, 15, and 20 wt % **[BP**_**Br**_**DMA]**_**2**_**·[BiBr**_**5**_**]**-PLA devices (Figures 4c and S20 and S21). The obtained *V*_PP_ of 26.2 V and *I*_PP_ of 10.63 μA are comparable to some of the best-performing PENGs based on two-component hybrid materials (Table S5).

Furthermore, to validate the utility of these devices under practical conditions, the voltage drops were recorded under different external load resistors ranging from 0.11 to 88 MΩ in the circuit. An increasing trend in the voltage drop (approaching the peak voltage) was observed while increasing load resistance until 22 MΩ and saturation in voltage drop was detected for higher load resistances (Figure S22). The peak power density (*P* = *VI*/*A*) was subsequently calculated at the entire range of load-resistances for the best performing 15 wt % **[BP**_**Br**_**DMA]**_**2**_**·[BiBr**_**5**_**]**-PLA device. The maximum peak power density was calculated to be 15.47 μW cm^–2^ at the optimal 1 MΩ resistance (Figure 4d). The cyclic stability measurements performed up to 3000 cycles showed retention in the voltage output, without any degradation, for the 15 wt % **[BP**_**Br**_**DMA]**_**2**_**·[BiBr**_**5**_**]**-PLA device (Figure S23).

**Figure 5:**
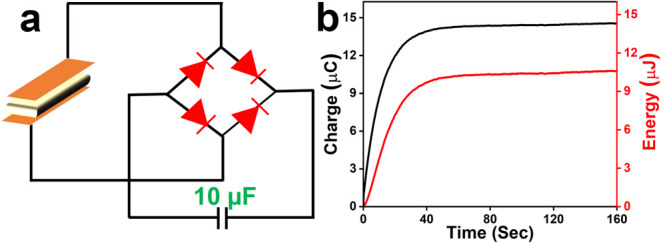
(a) Schematic of the four-diode bridge rectifier circuit utilized for capacitor charging experiments. (b) Charge and energy stored in a 10 μF capacitor by using the champion 15 wt % **[BP**_**Br**_**DMA]**_**2**_**·[BiBr**_**5**_**]**-PLA composite device.

The real-life PENG application of the best performing 15 wt % **[BP**_**Br**_**DMA]**_**2**_**·[BiBr**_**5**_**]**-PLA composite device was further examined by performing capacitor charging experiments using a 10 μF capacitor. A four-diode bridge rectifier circuit, which converts the AC signals into DC outputs, was kept in the circuit for charge storage (Figure 5a). The device stored a maximum charge of 14 μC in the 10 μF capacitor within 160 s (Figure 5b). The corresponding voltage and calculated energies were of the order of 1.45 V and 10 μJ, respectively (Figures 5b and S24).

## Conclusions

To summarize, we have successfully designed and synthesized a new ferroelectric complex belonging to the A_2_BiX_5_ subfamily of hybrid ammonium halogenobismuthates(III), **[BP**_**Br**_**DMA]**_**2**_**·[BiBr**_**5**_**]**. It crystallized in the polar noncentrosymmetric space group *Pna*2_1_ and showed a relative SHG efficiency of 0.039 compared to the standard KDP sample. The ferroelectric nature of **[BP**_**Br**_**DMA]**_**2**_**·[BiBr**_**5**_**]** was confirmed by *P*–*E* hysteresis loop measurements, which yielded a saturation polarization (*P*_s_) of 5.72 μC cm^–2^. The microscopic polarization domains in **[BP**_**Br**_**DMA]**_**2**_**·[BiBr**_**5**_**]** were visualized through PFM measurements. The PFM-spectroscopy performed at a single point on its topographic surface showed the characteristic phase-bias hysteresis and amplitude-bias butterfly loops. The implementation of **[BP**_**Br**_**DMA]**_**2**_**·[BiBr**_**5**_**]** for mechanical energy harvesting applications has been done by preparing its composite films in PLA polymer. A maximum peak-to-peak voltage of 26.2 V and a power density of 15.47 μW cm^–2^ has been recorded for the 15 wt % **[BP**_**Br**_**DMA]**_**2**_**·[BiBr**_**5**_**]**-PLA champion device. The harvested electrical energies, after rectification, could successfully be stored in a 10 μF capacitor, with charge saturation taking place within 160 s. These findings pave the way toward the design of new lead-free organic–inorganic hybrids using the benign Bi^3+^ ions and simple ammonium cations for use in next-generation electronic and optical devices.

## Supporting Information Available

Experimental procedures, synthesis, additional crystal structures, HS analyses, characterizations, dielectric analysis, piezoresponse force microscopic data, and piezoelectric energy harvesting outputs (PDF)
