# Supplementary material for: Ferroelectric Organic–Inorganic Hybrid Ammonium Halogenobismuthate(III) for Piezoelectric Energy Harvesting
Source: Inorg Chem. 2024 May 3;63(20):9245–51. doi: 10.1021/acs.inorgchem.4c00908 (PMC11110009; doi:10.1021/acs.inorgchem.4c00908)
Supplement: Supplementary file 1 — ic4c00908_si_001.pdf [file ic4c00908_si_001.pdf]

Supporting Information

Ferroelectric Organic-Inorganic Hybrid Ammonium Halogenobismuthate(III) for Piezoelectric Energy Harvesting

Namonarayan Meena,[a,†] Supriya Sahoo,[a,†] Nilotpal Deka,[a] Jan K. Zaręba,[c,*] Ramamoorthy Boomishankar[a,b,*]

[a]Department of Chemistry and [b]Centre for Energy Science, Indian Institute of Science Education and Research, Pune, Dr. Homi Bhabha Road, Pune – 411008, India

Email: boomi@iiserpune.ac.in

[c]Institute of Advanced Materials, Wrocław University of Science and Technology, 50-370 Wrocław, Poland

Email: jan.zareba@pwr.edu.pl

†N.M. and S.S. contributed equally to this paper

Table of contents

S.No.	Details	Page No.
1	Experimental Section	S2-S3
2	Synthesis and X-ray crystallographic information of **$BP_{Br}DMA·Br$** and **$[BP_{Br}DMA]_2·[BiBr_5]$**	S3-S6
3	Hirshfeld surface analysis data and characterizations	S7-S9
4	Dielectric, ferroelectric and PFM spectroscopic analysis	S9-S10
5	Preparation of **$[BP_{Br}DMA]_2·[BiBr_5]$**-PLA composites, characterizations and their Piezoelectric Energy Harvesting studies	S10-S14
6	References	S14

EXPERIMENTAL SECTION

General Remarks

4-Bromo-N,N-dimethylaniline was purchased from Merck, benzyl bromide was procured from Lobachemie, India and bismuth(II) oxide and hydrobromic acid were acquired from Avra Chemicals, India and directly employed without further purification. Thermogravimetric analyses were performed on the PerkinElmer STA-6000 analyzer, with a heating rate of 10 °C/min, and the differential scanning calorimetry (DSC) measurements using a TA Q20 differential scanning calorimeter, with heating and cooling rates of 10 °C/min. Both these measurements were performed under a dry nitrogen atmosphere. Melting points without correction were recorded on a Buchi M-560 melting point apparatus. The FT-IR spectra were obtained using the Bruker (Invenio R), covering the range of 400-4000 cm^{-1}. Powder X-ray diffraction (PXRD) data, in the 2θ range of 5 to 50º, were collected using the Bruker-D8 Advance X-ray diffractometer. The field-emission scanning electron microscopy (FE-SEM) images of all composite films (with different weight percentages of **$[BP_{Br}DMA]_2 \cdot [BiBr_5]$**) were recorded using the Zeiss ultra plus FE-SEM instrument with a minimum spatial resolution of 1 μm.

Synthesis of $BP_{Br}DMA \cdot Br$:

Benzyl bromide (0.5 ml, 4 mmol) in hexane was added to a stirred solution of 4-Bromo-N,N-dimethylaniline (800 mg, 4 mmol) in 5 ml of methanol [Scheme S1]. The resulting solution was stirred for 30 minutes and was kept for crystallization for 3 days to yield the bromide salt **$BP_{Br}DMA \cdot Br$**. Yield: 82 %. Melting point: 424-429 K. Anal. calcd. for **$BP_{Br}DMA \cdot Br$**: C 48.55; H 4.62; N 3.77 Found: C 48.45; H 4.60; N 3.75.

Synthesis of $[BP_{Br}DMA]_2 \cdot [BiBr_5]$:

The bromide salt **$BP_{Br}DMA \cdot Br$** (900 mg, 3 mmol) was dissolved in concentrated HBr (48% N, 2 mL) and subsequently added in a dropwise manner to a solution of Bi_2O_3 (705 mg, 1.5 mmol) in concentrated HBr (9 N, 4 mL) [Scheme S2]. The resulting solution was stirred for 30 minutes and the obtained yellow precipitate was collected by filtration. Yellow crystals of **$[BP_{Br}DMA]_2 \cdot [BiBr_5]$** suitable for single crystal X-ray diffraction analysis were obtained from its acetonitrile and acetone (1:1) solution after 7-8 days. Yield: 85 %. FT-IR data in KBr pellet (cm^{-1}): 2923, 1700, 1621, 1455, and 1111. Anal. calcd. for **$[BP_{Br}DMA]_2 \cdot [BiBr_5]$**: C 30.26; H 2.88; N 2.35. Found: C 30.21; H 2.85; N 2.30.

Single Crystal X-ray Diffraction Analysis:

The single-crystal X-ray diffraction data for compounds **$BP_{Br}DMA \cdot Br$** and **$[BP_{Br}DMA]_2 \cdot [BiBr_5]$** were collected on a Bruker Smart Apex Duo diffractometer with Mo Kα radiation (λ=0.71073 Å). The crystal structures were solved through the direct method and refined using full-matrix least-squares against F^2, employing the SHELXL-2014/7 program integrated into the Apex 3 software.[1] Anisotropic refinement was applied to all nonhydrogen atoms, while hydrogen atoms were constrained in geometric positions relative to their parent atoms.[2] The data shows some amount of residual electron densities that could be attributed to the presence of solvated molecules. Owing to the diffuse nature of solvated atoms (in the vicinity of bulky Bi and Br atoms), they could not be modeled appropriately even at 120 K. Hence, these were treated as diffuse contributions to the overall scattering and removed by the SQUEEZE/PLATON method for better refinement data. The volumes squeezed in the structure were found to be 540 $Å^3$, which approximately corresponds to 7 methanol molecules and 3 water molecules in the unit cell of **$[BP_{Br}DMA]_2 \cdot [BiBr_5]$** as determined from thermogravimetric analysis (TGA). Structural illustrations were generated using the DIAMOND-3.1 software.

Hirshfeld Surface Analysis:

The Crystal Explorer 3.1 program was employed to perform Hirshfeld surface analysis on **$[BP_{Br}DMA]_2 \cdot [BiBr_5]$.** For Hirshfeld analysis, the single-crystal X-ray crystallographic information file (CIF) was utilized and the diverse interactions present in **$[BP_{Br}DMA]_2 \cdot [BiBr_5]$** were visualized. The resulting 3D color mapping images illustrate the surface characteristics of the compound **$[BP_{Br}DMA]_2 \cdot [BiBr_5]$**; red: intense interactions, blue: medium interactions, and white: weak interactions. A 2D fingerprint plot, essentially a histogram, was generated by compiling distances between atoms closest to the interior (d_i) and exterior (d_e) of the Hirshfeld surface. The plot featured various contours represented by blue and grey colors, offering valuable insights into the various molecular interactions present in the molecule.

Nonlinear Optical Measurements:

The Kurtz-Perry powder tests were conducted using a Coherent Astrella Ti: Sapphire regenerative amplifier (RA) to generate femtosecond laser pulses with a repetition rate of 1 kHz. These laser pulses were directed through a wavelength-tuneable Topaz Prime Vis-NIR optical parametric amplifier (OPA) to achieve the desired wavelength of 1300 nm. The laser beams, with a fluence of 0.20 mJ cm^{-2} at 1300 nm, were unfocused. The SHG relative efficiency of **$[BP_{Br}DMA]_2 \cdot [BiBr_5]$** was determined through the Kurtz-Perry powder method, utilizing potassium dihydrogen phosphate (KDP) as the SHG reference. Microcrystals of **$[BP_{Br}DMA]_2 \cdot [BiBr_5]$** and KDP were

independently ground and sieved using an Aldrich mini-sieve set to obtain a microcrystal size fraction of 250−177 μm. The laser beam was directed at the samples at an angle of 45° degrees, and the diffused SHG spectra were recorded by an Ocean Optics Flame T spectrograph after suppressing the scattered pumping radiation with a 750 nm short-pass dielectric filter.

Ferroelectric, Dielectric and Piezoelectric Measurements:

To investigate the ferroelectric properties of **$[BP_{Br}DMA]_2\cdot[BiBr_5]$**, *P-E* hysteresis loop measurements were conducted on a thin film sample of approximately 2.21 μm thickness drop-casted on an Indium tin oxide (ITO)-coated glass surface, and Gallium Indium eutectic was used to the make top contact. These measurements were performed using the aixACCT TF-2000E model hysteresis loop analyzer. The experiments were conducted by applying the dynamic leakage current compensation (DLCC) mode to reduce the contributions from non-hysteretic components of the loop.

The dielectric permittivity of **$[BP_{Br}DMA]_2\cdot[BiBr_5]$** was measured on its powder-pressed pellet sample. The measurements were conducted using the Solartron Analytical 1260 model Impedance Analyzer combined with a Dielectric Interface model 1296A. The sample was placed in a Janis 129610A cryostat sample holder, and the temperature was controlled using a Lakeshore 336 model temperature controller.

Piezoresponse Force Microscopy (PFM) Characterizations:

The PFM measurements were conducted using the Asylum Research MFP-3D atomic force microscopy (AFM) system for a drop-casted thin film of **$[BP_{Br}DMA]_2\cdot[BiBr_5]$** on ITO-coated glass surface. A contact mode AFM experiment was carried out, utilizing RMN-12PT300B cantilever probes with a spring constant of 1.12 N m^{-1} and a tip diameter of less than 8 nm to measure the piezoresponse of the crystal films. Vertical-PFM experiments were employed to acquire PFM data, with the bottom electrode being grounded, while an AC voltage was applied to the conductive AFM tip. PFM images were collected at the resonance frequency of 300 +/- 20 kHz with an applied bias of 60 and 80 V. The measurements were performed using the dual AC resonance tracking (DART) mode of the PFM. The switching ability of the domains of the single crystal located on the thin film was recorded by the application of external DC bias of ±120 using the PFM tip in contact mode.

General Procedure for the Preparation of Polymer Composite Films and Devices:

To fabricate composite films of **$[BP_{Br}DMA]_2\cdot[BiBr_5]$**, different proportions (5, 10, 15, and 20 wt%) of ferroelectric crystallites were dispersed in a nonpiezoelectric, biodegradable polylactic acid polymer (PLA) dissolved in chloroform ($CHCl_3$). The solutions underwent mechanical stirring at 50 ˚C for 15 minutes, followed by vortex mixing for 15 minutes to ensure homogeneity. Subsequently, these solutions were poured onto a glass slide and left undisturbed to air-dry at room temperature for 8 hours. The resulting composite films were carefully peeled off from the glass slide, and copper conductive adhesive tapes were affixed to both sides to establish electrical contacts. To complete the architecture, the devices were covered with Kapton tapes. A pristine PLA polymer film encapsulated with Kapton tape was also prepared and subjected to similar experimental conditions for comparative analysis.

Piezoelectric Energy Harvesting and Storage Measurements:

An in-house-designed periodic impact set-up was employed to conduct mechanical energy harvesting experiments, applying an impact force of 21 N. The Tektronix 2024 Mixed Signal Oscilloscope, featuring an input impedance of 1 MΩ, was utilized for measuring output voltages and currents. The test devices, with a thickness of approximately 0.5 mm and an active area of 300 mm^2, were subjected to examination. In order to assess the energy storage capabilities of the devices during impact measurements, a 10 μF capacitor was connected to the device using a full-wave bridge four-diode circuit.

Scheme 1

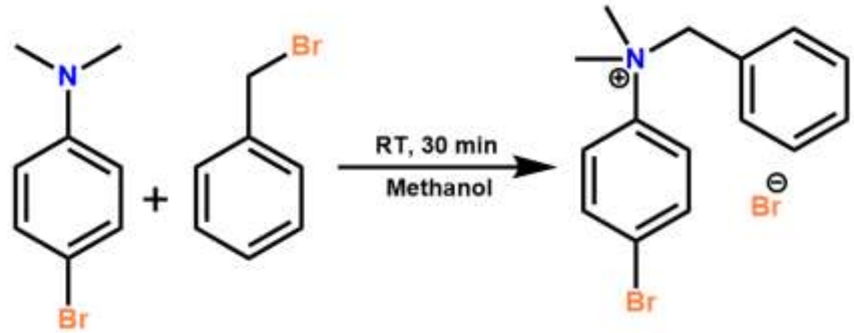

Scheme S1. Schematic showing the synthesis of **$BP_{Br}DMA\cdot Br$**.

Scheme 2

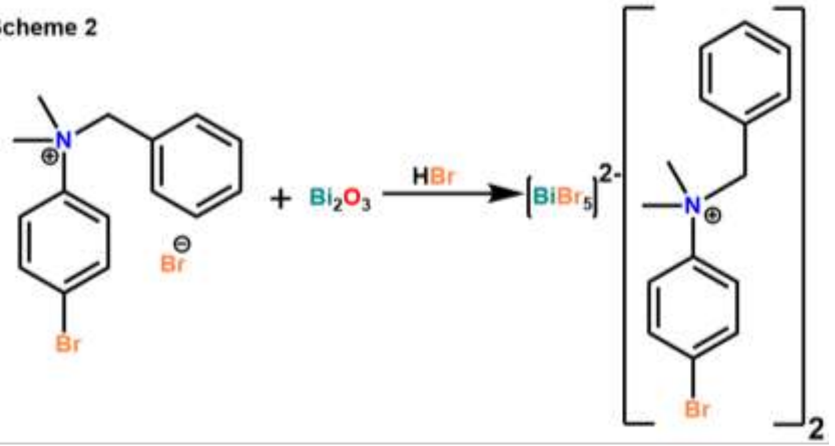

Scheme S2. Schematic showing the synthesis **$[BP_{Br}DMA]_2\cdot[BiBr_5]$.**

Table S1. X-ray Crystallographic data for **$BP_{Br}DMA\cdot Br$** and **$[BP_{Br}DMA]_2\cdot[BiBr_5]$**.

Crystallographic details	$BP_{Br}DMA\cdot Br$ (150 K)	$[BP_{Br}DMA]_2\cdot[BiBr_5]$ (120 K)	$[BP_{Br}DMA]_2\cdot[BiBr_5]$ (298 K)
Chemical formula	$C_{15}H_{17}Br_2N$	$C_{30}H_{34}BiBr_7N_2$	$C_{30}H_{34}BiBr_7N_2$
Formula weight (g/mol)	371.11	1190.94	1190.94
Temperature	150(2)	120(2)	298(2)
Crystal system	Monoclinic	Orthorhombic	Orthorhombic
Space group	$P2_1/n$	$Pna2_1$	$Pna2_1$
a (Å); α (°)	10.410(5); 90	17.906(8); 90	18.20(3); 90
b (Å); β (°)	9.803(5); 93.854(12)	24.130(11); 90	24.35(4); 90
c (Å); γ (°)	14.302(7); 90	9.071(4); 90	9.170(14); 90
V (Å^3); Z	1456.2(13); 4	3919(3); 4	4063(10); 4
ρ (calc.) g cm^{-3}	1.693	2.018	1.947
μ (Mo K_α) mm^{-1}	5.549	11.655	11.242
$2\theta_{max}$ (°)	25.087	25.264	25.314
R(int)	0.1682	0.2342	0.3413
Completeness to θ	99.2	99.5	99.7
Data / param.	2568/165	7035/317	7297/317
GOF	1.017	0.920	0.989
R1 [F>4σ(F)]	0.0435	0.0500	0.0666
wR2 (all data)	0.0956	0.1244	0.1705
max. peak/hole (e.Å^{-3})	0.473/0.785	1.137/1.775	1.085/1.650

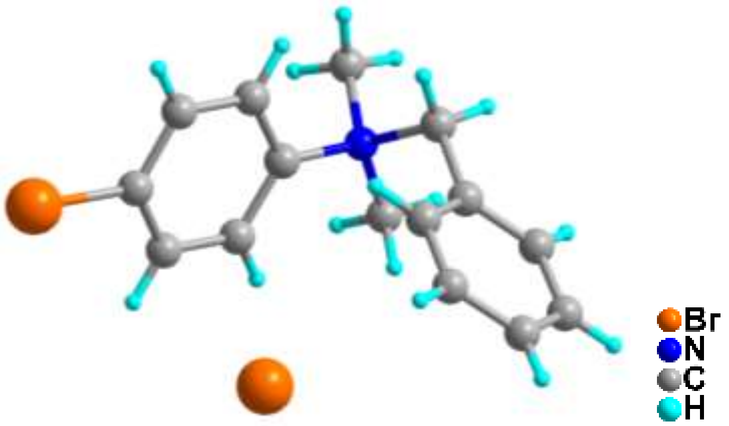

Figure S1: Asymmetric unit of **BP$_{Br}$DMA·Br** at 150 K.

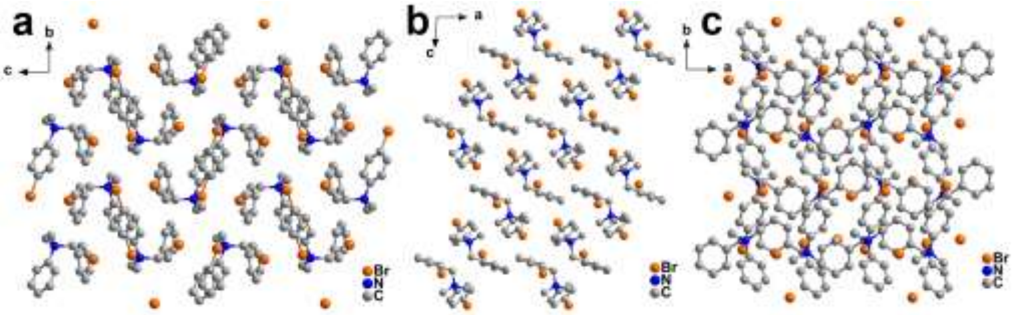

Figure S2. The 2 x 2 packing diagram of **BP$_{Br}$DMA·Br** along (a) *a*-axis, (b) *b*-axis (c) *c*-axis at 150 K.

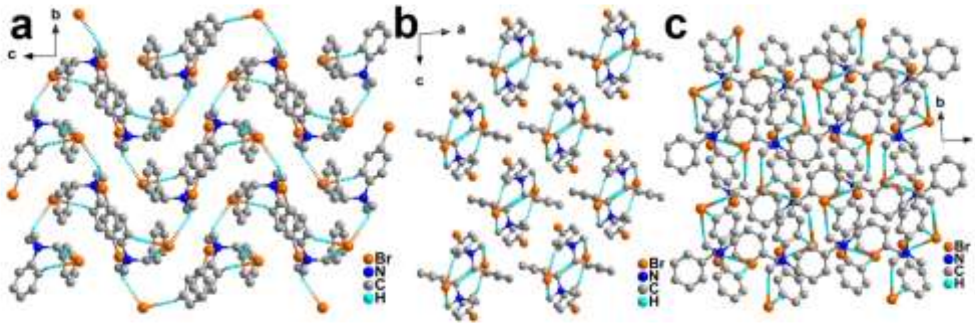

Figure S3. The C-H···Br hydrogen bonding interactions in **BP$_{Br}$DMA·Br** along (a) *a*-axis, (b) *b*-axis (c) *c*-axis at 150 K.

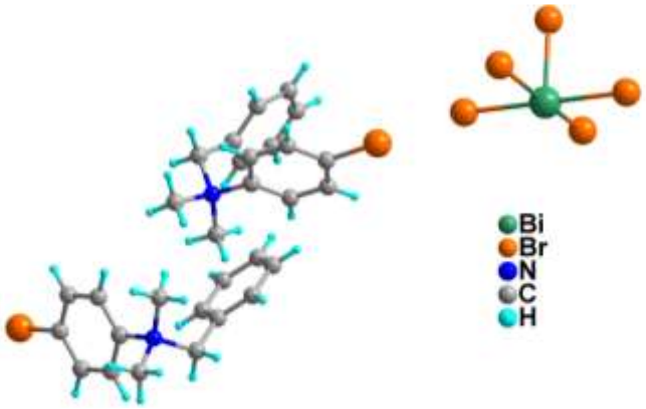

Figure S4: Asymmetric unit of **[BP$_{Br}$DMA]$_2$·[BiBr$_5$]** at 120 K.

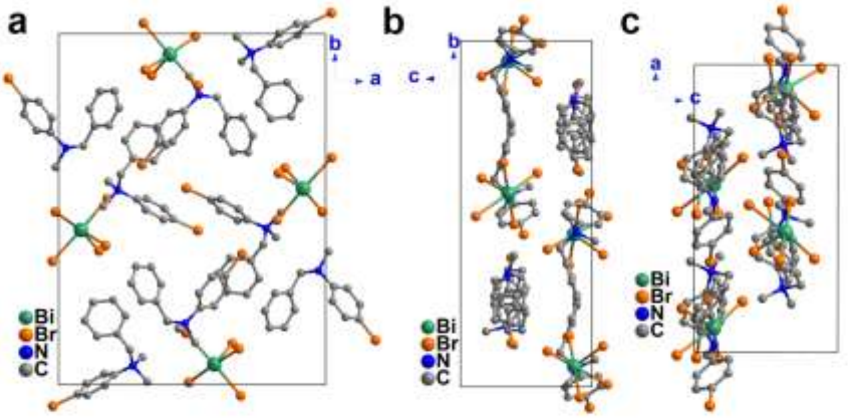

Figure S5. The packing diagram of **[BP$_{Br}$DMA]$_2$·[BiBr$_5$]** along (a) *c*-axis, (b) *a*-axis (c) *b*-axis at 120 K.

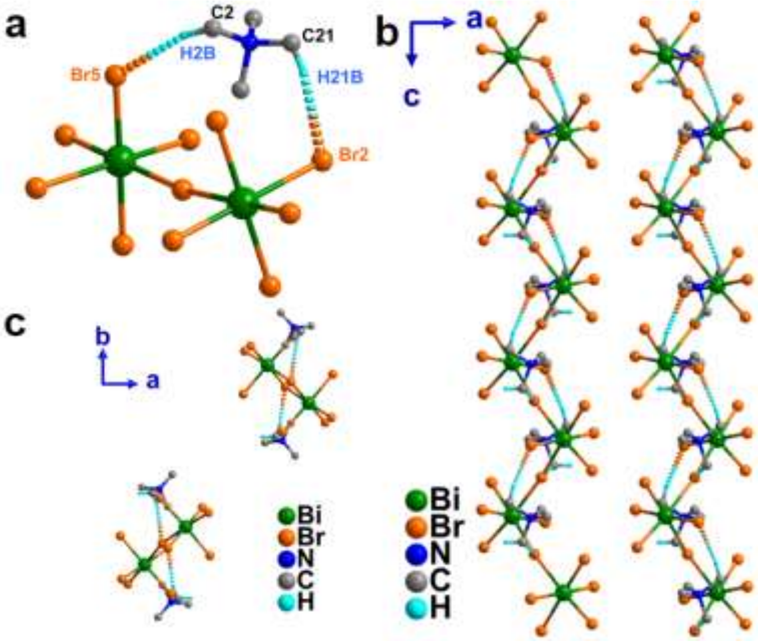

Figure S6. The C-H···Br hydrogen bonding interactions in **[BP$_{Br}$DMA]$_2$·[BiBr$_5$]** at 120 K along (a) *b*-axis and (b) *c*-axis.

Table S2. Hydrogen bonding parameters for **[BP$_{Br}$DMA]$_2$·[BiBr$_5$]** at 120 K.

D-H···A	d(H···A) Å	d(D-A) Å	<(DHA)˚	Symmetry transformations to generate equivalent atoms
C(2)-H(2B)···Br(5)	2.7364(108) Å	3.7022(818) Å	164.497(4662)˚	0.5-x, -0.5+y, -0.5+z
C(21)-H(21Bs)···Br(2)	2.8613(121) Å	3.8069(953) Å	165.056(5554)˚	0.5-x, -0.5+y, -0.5+z

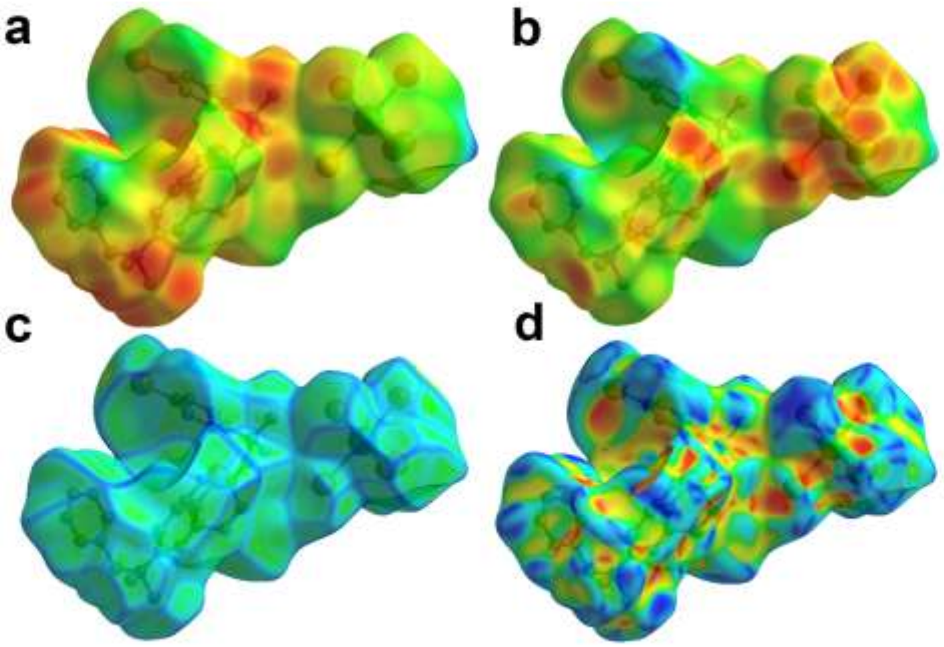

Figure S7. The 3D color mapping surfaces derived from the Hirshfeld surface analysis of **[BP$_{Br}$DMA]$_2$·[BiBr$_5$]** (120 K) showing (a) d_i, (b) d_e, (c) curvedness, and (d) shape index.

Table S3. Hirshfeld surface analysis of **[BP$_{Br}$DMA]$_2$·[BiBr$_5$]** at 120 K.

Temperature	Surface Property	Range (Minimum/Maximum)	Globularity and Asphericity	Surface Volume and Area
**120 K**	d_i	0.980/4.099	0.602 and 0.538	962.97 Å^3 and 783.65 Å^2
	d_e	0.980/3.688		
	d_{norm}	-0.382/2.781		
	Shape index	-0.997/0.998		
	Curvedness	-3.904/0.575		

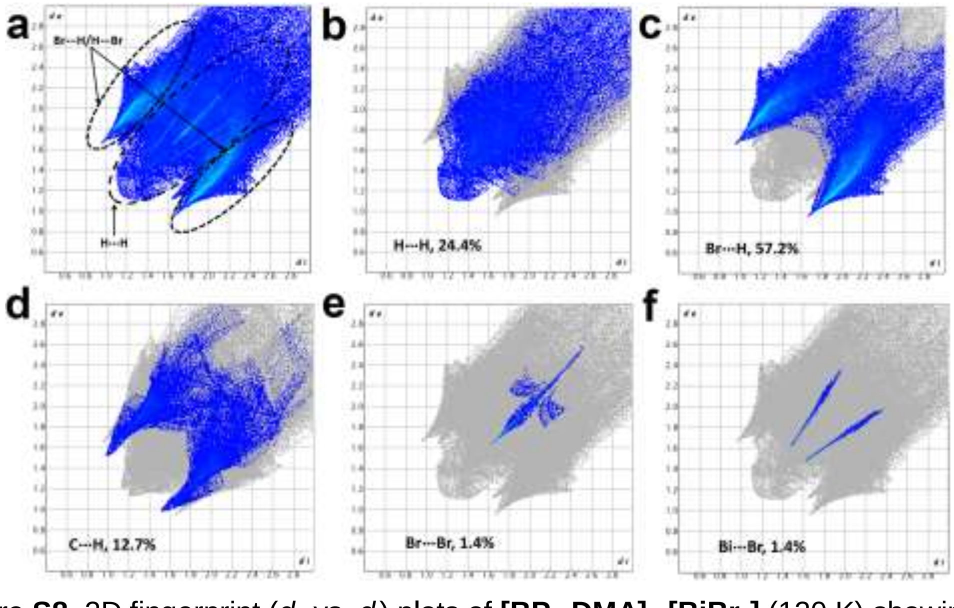

Figure S8. 2D fingerprint (d_e vs. d_i) plots of **[BP$_{Br}$DMA]$_2$·[BiBr$_5$]** (120 K) showing the percentages of (a) all interactions, (b) H···H, (c) Br···H/H···Br, (c) C···H/H···C, (d) Br···Br, and (f) Bi···Br/Br···Bi interactions.

Table S4. Percentage interactions present in **[BP$_{Br}$DMA]$_2$·[BiBr$_5$]** at 120 K.

Atom	Bi	Br	N	C	H	Total
Bi	-	0.7	-	-	0.4	1.1
Br	0.6	1.4	-	1.1	32.9	36.0
C	-	1.0	-	0.5	6.8	8.3
H	0.0	24.3	-	5.9	24.4	54.7
N	-	-	-	-	-	0.0
Total	0.6	27.5	0.0	7.5	64.4	100

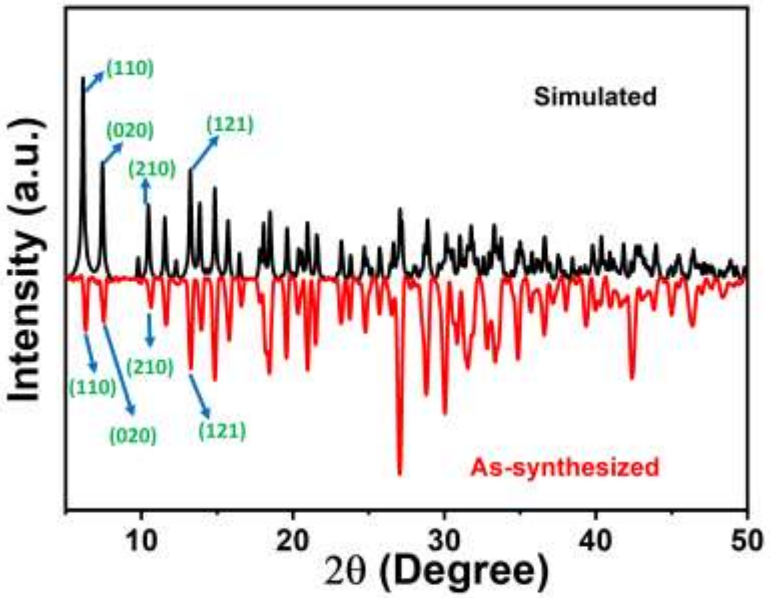

Figure S9. The room temperature PXRD profile of **$[BP_{Br}DMA]_2·[BiBr_5]$** along with its simulated profile from the 298 K SCXRD data.

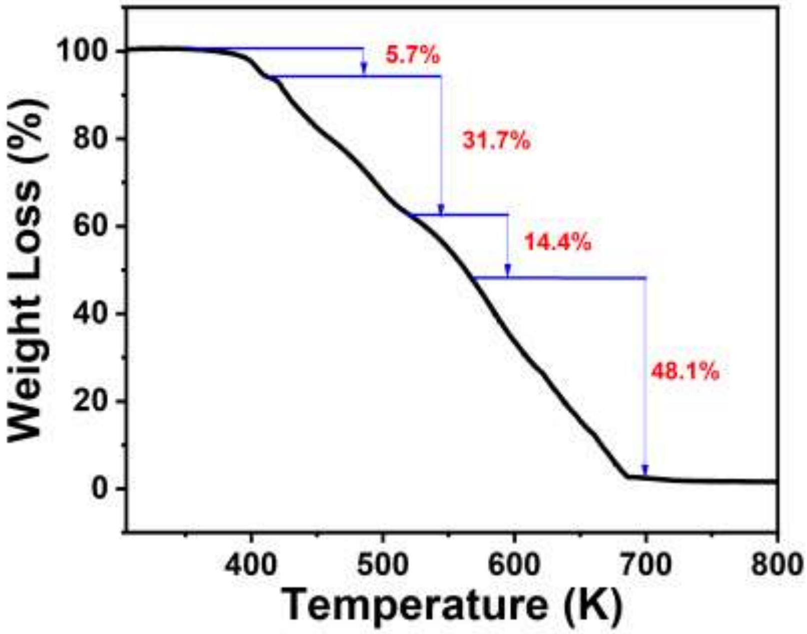

Figure S10. The thermogravimetric and differential thermal analysis profiles of **$[BP_{Br}DMA]_2·[BiBr_5]$**

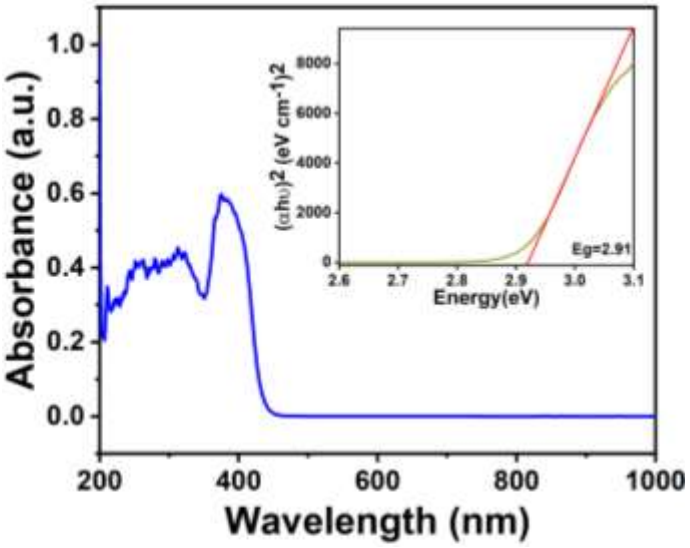

Figure S11. The UV-Vis diffuse reflectance spectrum of **$[BP_{Br}DMA]_2\cdot[BiBr_5]$**. The corresponding Tauc plot is displayed in the inset.

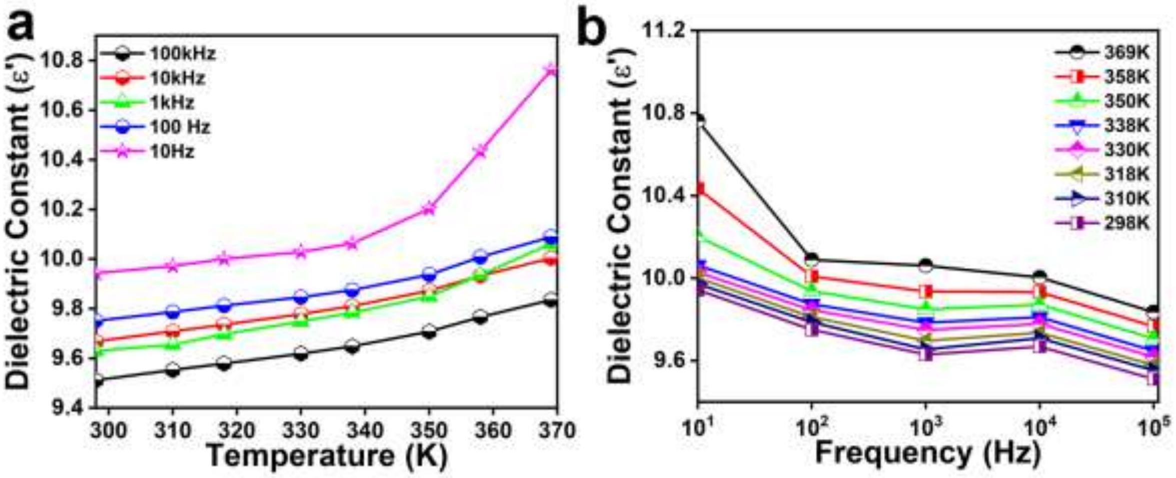

Figure S12. The (a) temperature-dependent (b) frequency-dependent dielectric permittivity plots of **$[BP_{Br}DMA]_2\cdot[BiBr_5]$**.

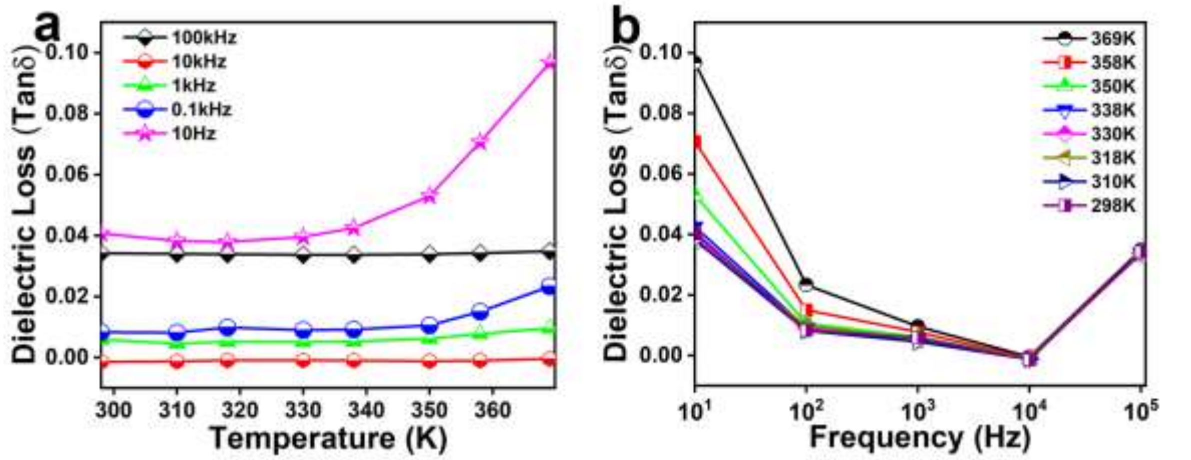

Figure S13. The (a) temperature-dependent (b) frequency-dependent dielectric loss profiles of **$[BP_{Br}DMA]_2\cdot[BiBr_5]$**.

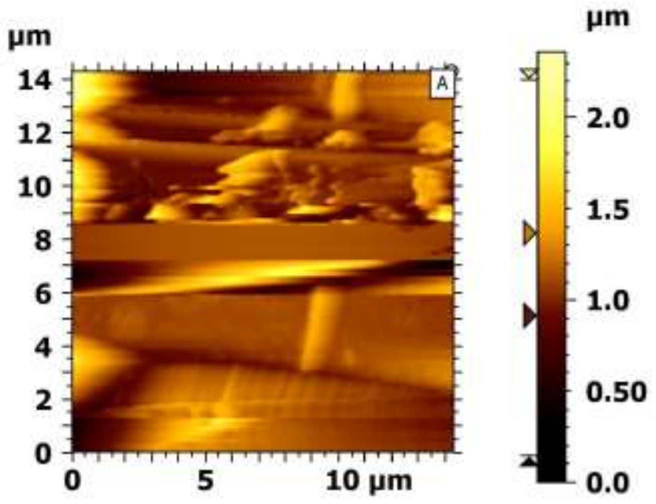

Figure S14. The atomic force microscopy (AFM) image of drop casted thin film sample of **$[BP_{Br}DMA]_2{\cdot}[BiBr_5]$** on ITO-coated glass substrate.

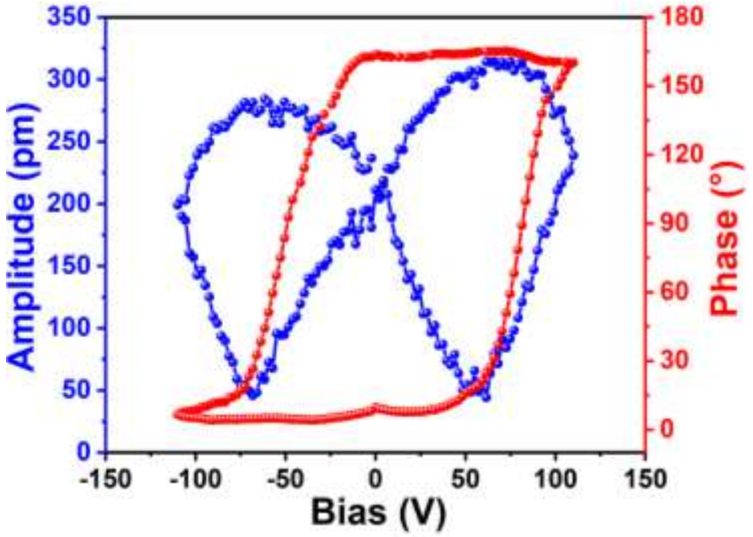

Figure S15. The off-state PFM amplitude-bias butterfly and phase-bias hysteresis loops for **$[BP_{Br}DMA]_2{\cdot}[BiBr_5]$**.

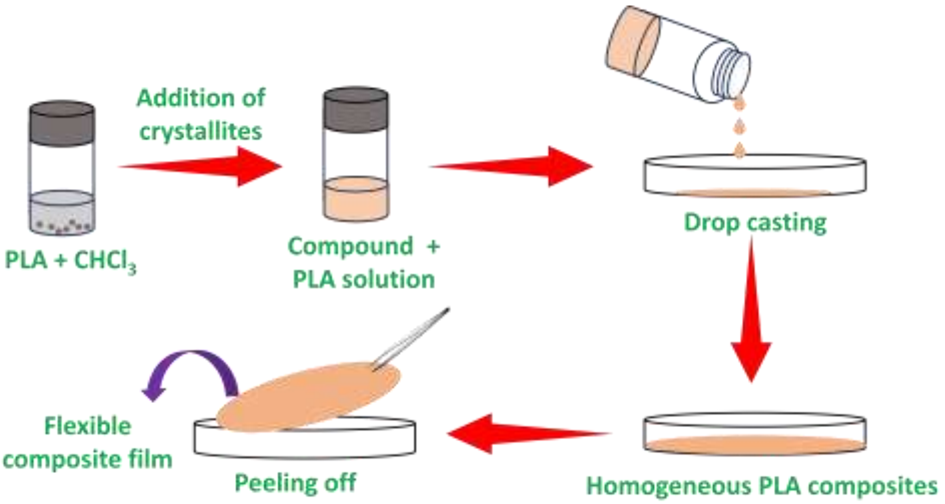

Figure S16. Schematic for the preparation of **$[BP_{Br}DMA]_2{\cdot}[BiBr_5]$**-PLA composite films.

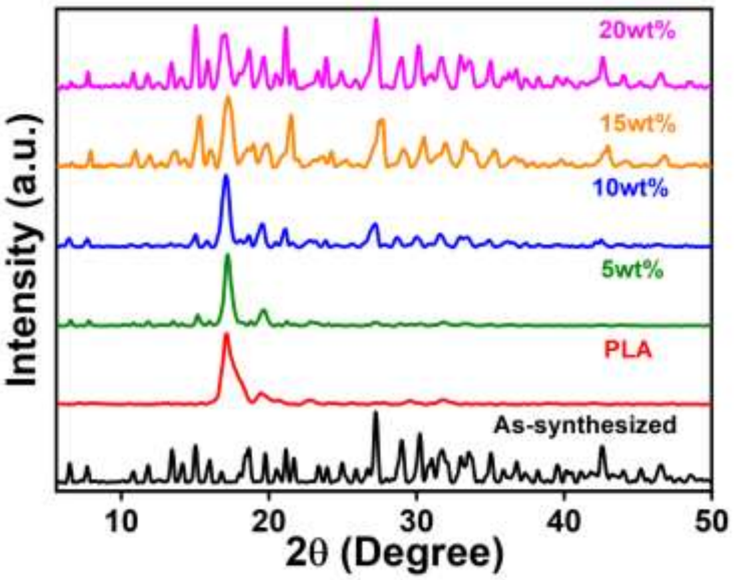

Figure S17. The PXRD profiles of **[BP_{Br}DMA]$_2$·[$BiBr_5$]**-PLA composites and their comparison with neat PLA and **[BP_{Br}DMA]$_2$·[$BiBr_5$]**.

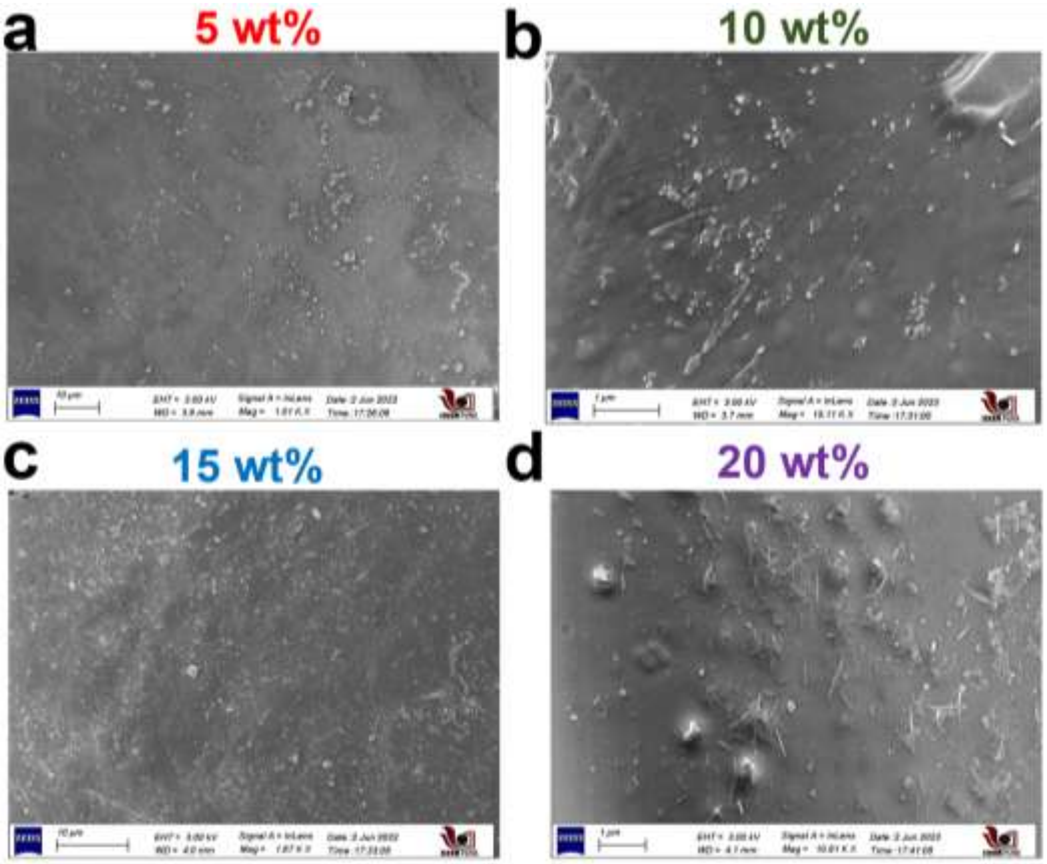

Figure S18. The FE-SEM images of 5, 10, 15, and 20 wt% **[BP_{Br}DMA]$_2$·[$BiBr_5$]**-PLA composite films.

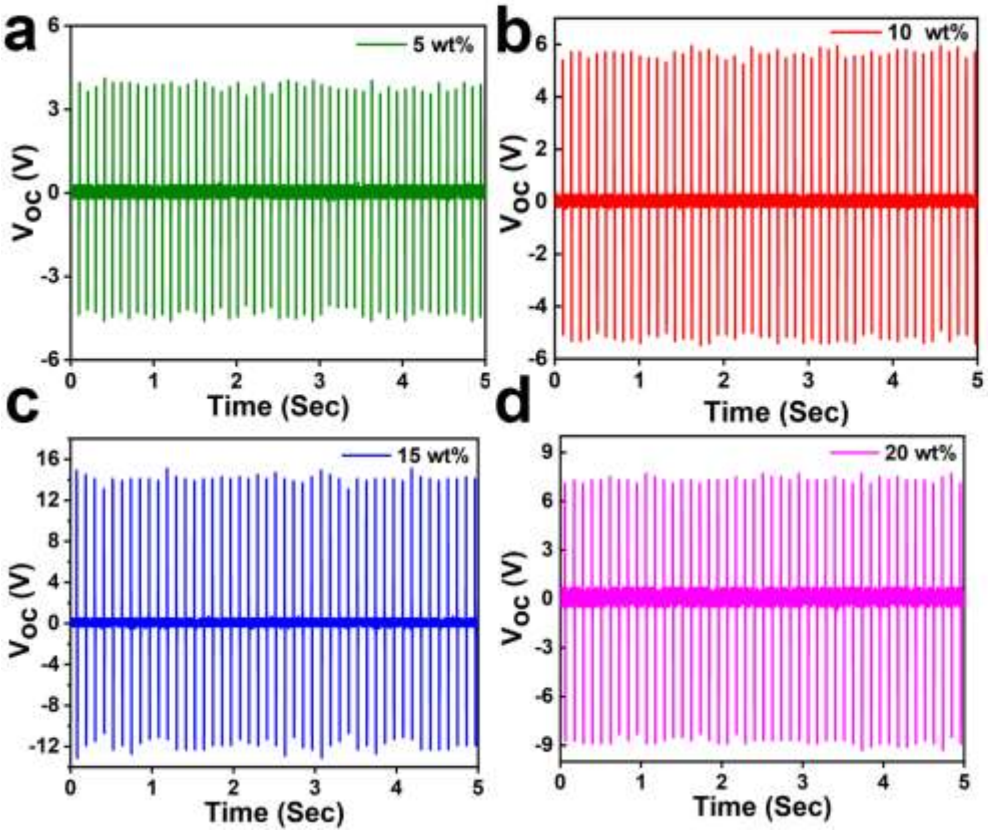

Figure S19. The open-circuit peak-to-peak voltage (V_{PP}) profiles of **$[BP_{Br}DMA]_2$·$[BiBr_5]$**-PLA composite devices.

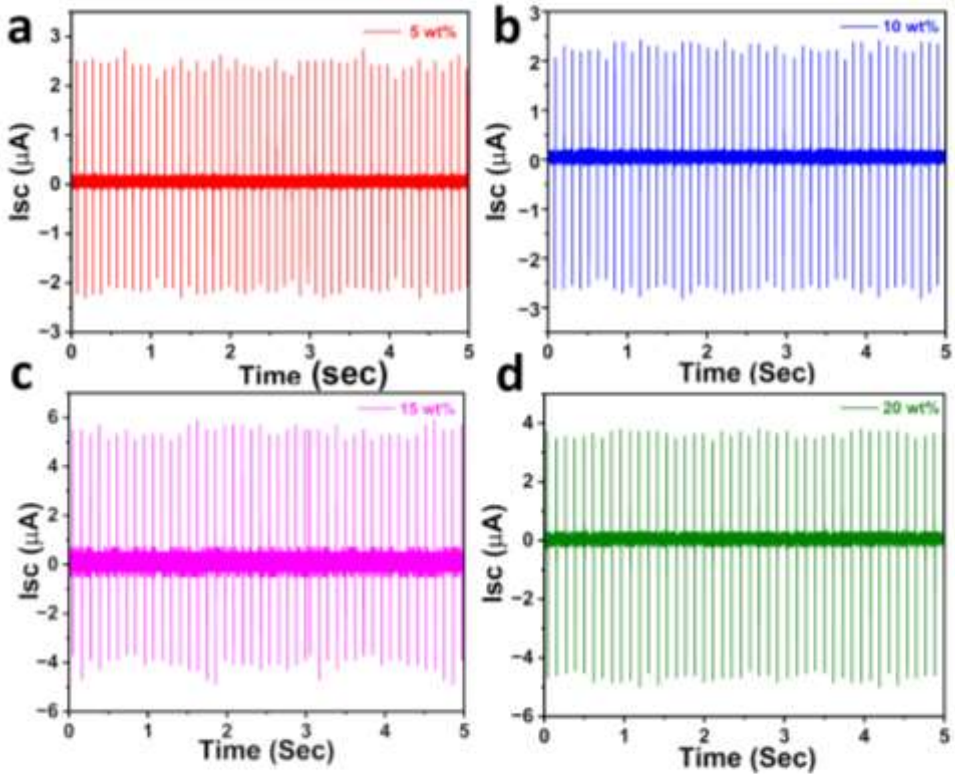

Figure S20. The output peak-to-peak current (I_{PP}) profile as calculated from the voltage drop obtained by attaching a 1 MΩ resistor across the circuit for the **$[BP_{Br}DMA]_2$·$[BiBr_5]$**-PLA composite devices.

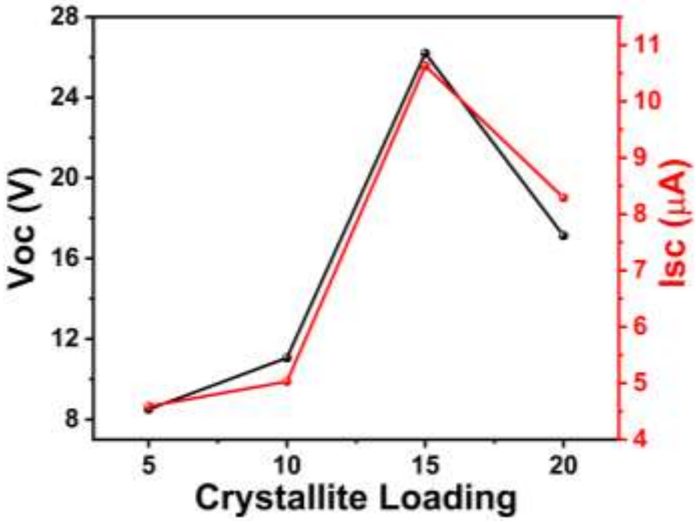

Figure S21. Comparative diagram showing the observed trends in V_{PP} and I_{PP} values of **$[BP_{Br}DMA]_2 \cdot [BiBr_5]$**-PLA composite devices.

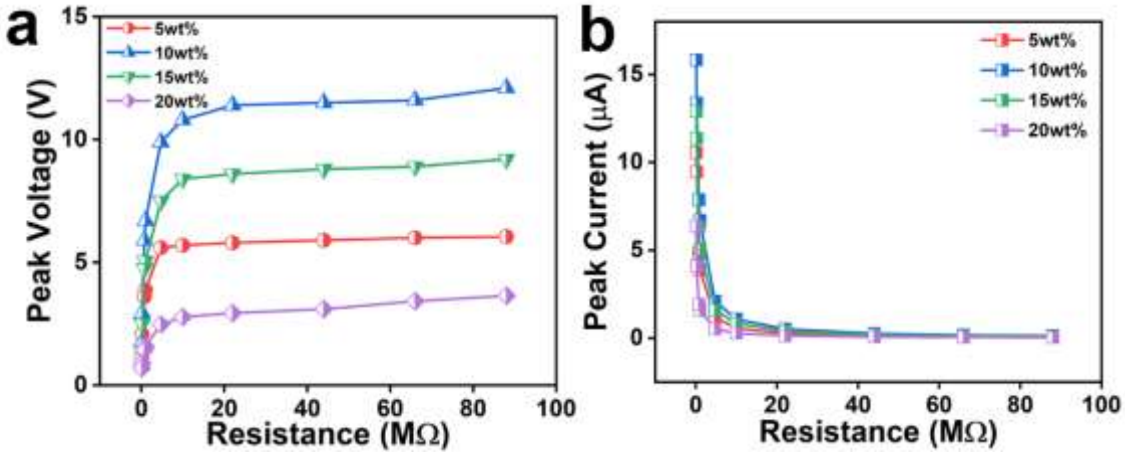

Figure S22. The comparative peak (a) voltage drop and (b) current data for all the **$[BP_{Br}DMA]_2 \cdot [BiBr_5]$**-PLA composite devices under various load resistances.

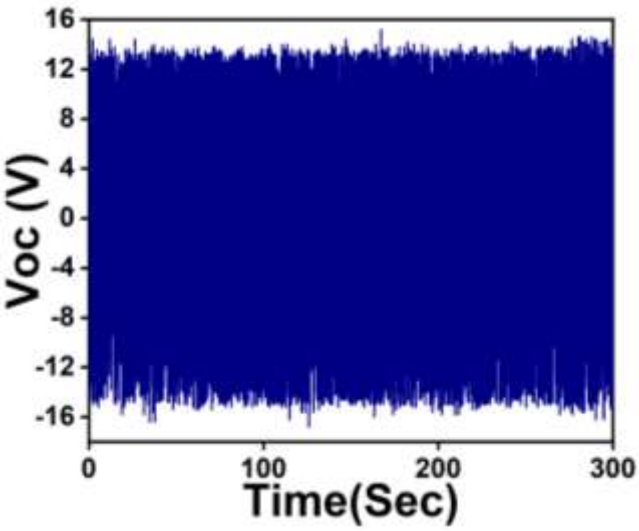

Figure S23. The cyclic stability test of 15 wt% **$[BP_{Br}DMA]_2 \cdot [BiBr_5]$**-PLA showing the retention of V_{PP} up to 3000 cycles.

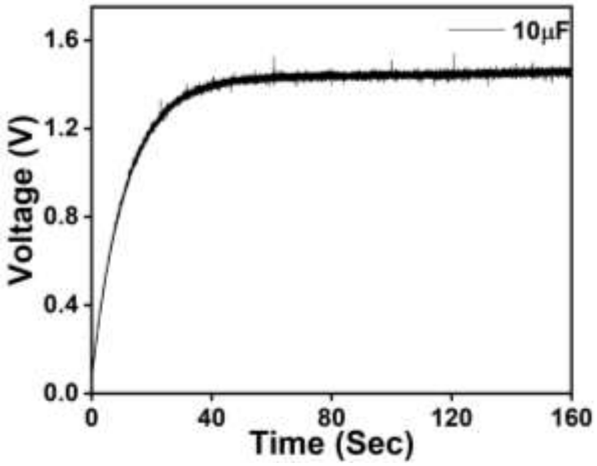

Figure S24. Voltage accumulated in a 10 µF capacitor by utilizing the 15 wt% **$[BP_{Br}DMA]_2$·$[BiBr_5]$**-PLA composite devise.

Table S5. Comparison of output device performances of known hybrid composite energy harvesters.

Hybrid Composite Devices	Output Voltages	Power/Power density	Active area	References
$MAPbI_3$-PVDF	9.43	-	1 x 1 cm^2	3
$MAPbBr_3$-PVDF	5	0.28 µW cm^{-2}	2.4 x 1.5 cm^2	4
$MAPbI_3$-PDMS	1.0	-	1 x 1 cm^2	5
$FAPbBr_3$–PDMS	4	-	1 x 1 cm^2	6
$CsPbBr_3$/PVDF	10.3	3.31 µW	1 x 1 cm^2	7
PVDF–PLLA–SnO_2 NF–$MAPbI_3$	4.82	-	0.25 x 0.25 cm^2	8
SnO_2 NF–$MAPbI_3$	1.02	-	0.25 x 0.25 cm^2	8
$[BnNMe_3]_2CdBr_4$/PDMS	52.9	13.8 µW cm^{-2}	3 x 3 cm^2	9
$[BnNMe_2{}^nPr]_2CdBr_4$/PDMS	63.8	37.1 µW cm^{-2}	3 x 3 cm^2	9
(TMFM)$FeBr_4$	2.2	-	-	10
$[Ph_2({}^iPrNH)_2P]_3[Fe(CN)_6]$-TPU	13.57	6.03 µW cm^{-2}	1.3 x 3 cm^2	11
$[Me(Ph)_3P]_3[Bi_2Br_9]$-PDMS	22.9	7 µW cm^{-2}	1.5x1.5 cm^2	12
$[Ph_3MeP]_4[Ni(NCS)_6]$/TPU	19.29	2.51 mW cm^{-3}	1.3 x 3 cm^2	13
$[Ph_3PMe]_4[CuCl_4]$-TPU	25	14.1 µW cm^{-2}	1.2 x 3 cm^2	14
$\{[{}^sCH(MePh)(Me)NH_3][BiBr_5]\}_n$-PLA	10.4	5.26 µW Cm^{-2}	1.2 x 3 cm^2	15
**$[BP_{Br}DMA]_2$·$[BiBr_5]$-PLA**	**26.2**	**15.47 µW cm^{-2}**	**1x 3 cm^2**	**This Work**

Note: $MAPbI_3$ = methylammonium lead iodide; PVDF = polyvinylidene difluoride; PDMS = polydimethysiloxane; $FAPbBr_3$ = formamidinium lead bromide; PLLA = poly(L-lactic acid); SnO_2 = tin oxide; NF = nanofiber; $[BnNMe_3]_2CdBr_4$ = N,N,N-trimethyl-1-phenylmethanaminium cadmium(II) bromide; $[BnNMe_2{}^nPr]_2CdBr_4$ = N-benzyl-N,N-dimethylpropan-1-aminium cadmium(II) bromide; (TMFM)$FeBr_4$ = trimethylfluoromethylammonium iron(III)bromide; TPU = thermoplastic polyurethane; Ph = Phenyl, Bn = Benzyl, ^{i}Pr = isopropyl, Me = Methyl.

References:

1. Sheldrick, G. M., A short history of SHELX. *Acta Crystallogr. A: Foundations of Crystallography* **2008,** *64*, 112-122.
2. Spek, A. L., Structure validation in chemical crystallography. *Acta Crystallogr. D: Biological Crystallography* **2009,** *65*, 148-155.
3. Jella, V.; Ippili, S.; Eom, J.-H.; Choi, J.; Yoon, S.-G., Enhanced output performance of a flexible piezoelectric energy harvester based on stable $MAPbI_3$-PVDF composite films. *Nano Energy* **2018,** *53*, 46-56.
4. Sultana, A.; Alam, M. M.; Sadhukhan, P.; Ghorai, U. K.; Das, S.; Middya, T. R.; Mandal, D., Organo-lead halide perovskite regulated green light emitting poly (vinylidene fluoride) electrospun nanofiber mat and its potential utility for ambient mechanical energy harvesting application. *Nano Energy* **2018,** *49*, 380-392.
5. Kim, Y.-J.; Dang, T.-V.; Choi, H.-J.; Park, B.-J.; Eom, J.-H.; Song, H.-A.; Seol, D.; Kim, Y.; Shin, S.-H.; Nah, J., Piezoelectric properties of $CH_3NH_3PbI_3$ perovskite thin films and their applications in piezoelectric generators. *J. Mater. Chem. A* **2016,** *4*, 756-763.
6. Ding, R.; Liu, H.; Zhang, X.; Xiao, J.; Kishor, R.; Sun, H.; Zhu, B.; Chen, G.; Gao, F.; Feng, X., Flexible piezoelectric nanocomposite generators based on formamidinium lead halide perovskite nanoparticles. *Adv. Funct. Mater.* **2016,** *26*, 7708-7716.
7. Li, Y.; Xu, M.-h.; Xia, Y.-s.; Wu, J.-m.; Sun, X.-k.; Wang, S.; Hu, G.-h.; Xiong, C.-x., Multilayer assembly of electrospun/electrosprayed PVDF-based nanofibers and beads with enhanced piezoelectricity and high sensitivity. *J. Chem. Eng.* **2020,** *388*, 124205.
8. Tusiime, R.; Zabihi, F.; Tebyetekerwa, M.; Yousry, Y. M.; Wu, Y.; Eslamian, M.; Yang, S.; Ramakrishna, S.; Yu, M.; Zhang, H., High stress-driven voltages in net-like layer-supported organic–inorganic perovskites. *J. Mater. Chem. C* **2020,** *8*, 2643-2658.
9. Deswal, S.; Singh, S. K.; Rambabu, P.; Kulkarni, P.; Vaitheeswaran, G.; Praveenkumar, B.; Ogale, S.; Boomishankar, R., Flexible Composite Energy Harvesters from Ferroelectric A_2MX_4-Type Hybrid Halogenometallates. *Chem. Mater.* **2019,** *31*, 4545-4552.
10. Zhang, Y.; Song, X.-J.; Zhang, Z.-X.; Fu, D.-W.; Xiong, R.-G., Piezoelectric energy harvesting based on multiaxial ferroelectrics by precise molecular design. *Matter* **2020,** *2*, 697-710.
11. Vijayakanth, T.; Sahoo, S.; Kothavade, P.; Bhan Sharma, V.; Kabra, D.; Zaręba, J. K.; Shanmuganathan, K.; Boomishankar, R., A Ferroelectric Aminophosphonium Cyanoferrate with a Large Electrostrictive Coefficient as a Piezoelectric Nanogenerator. *Angew. Chem. Int. Ed.* **2023,** *62*, e202214984.
12. Deswal, S.; Panday, R.; Naphade, D. R.; Dixit, P.; Praveenkumar, B.; Zaręba, J. K.; Anthopoulos, T. D.; Ogale, S.; Boomishankar, R., Efficient Piezoelectric Energy Harvesting from a Discrete Hybrid Bismuth Bromide Ferroelectric Templated by Phosphonium Cation. *Chem. Eur. J.* **2022,** *28*, e202200751.
13. Vijayakanth, T.; Ram, F.; Praveenkumar, B.; Shanmuganathan, K.; Boomishankar, R., Piezoelectric Energy Harvesting from a Ferroelectric Hybrid Salt $[Ph_3MeP]_4[Ni(NCS)_6]$ Embedded in a Polymer Matrix. *Angew. Chem. Int. Ed.* **2020,** *59*, 10368-10373.
14. Sahoo, S.; Vijayakanth, T.; Kothavade, P.; Dixit, P.; Zaręba, J. K.; Shanmuganathan, K.; Boomishankar, R., Ferroelectricity and Piezoelectric Energy Harvesting of Hybrid A_2BX_4-Type Halogenocuprates Stabilized by Phosphonium Cations. *ACS Mater. Au.* **2022,** *2*, 124-131.
15. Sahoo, S.; Deka, N.; Boomishankar, R., Piezoelectric energy harvesting of a bismuth halide perovskite stabilised by chiral ammonium cations. *CrystEngComm* **2022,** *24*, 6172-6177.
